# Cardioprotective effects of the extracellular chaperone clusterin in acute myocardial infarction

**DOI:** 10.1111/eci.70076

**Published:** 2025-05-26

**Authors:** Louwana Allawa, Antoine Poirier, Pascale Pignon, Justine Beaumont, Lucie Lebeau, Agnès Barbelivien, Thomas Bochaton, Céline Beauvillain, Daniel Henrion, Yves Delneste, Pascale Jeannin, Fabrice Prunier, Sophie Tamareille

**Affiliations:** ^1^ CNRS, Inserm, Laboratoire MITOVASC, SFR ICAT Univ Angers Angers France; ^2^ Service de Cardiologie CHU Angers Angers France; ^3^ Nantes Université, CNRS, Inserm, CRCI^2^NA, SFR ICAT Univ Angers Angers France; ^4^ Service d'Explorations Fonctionnelles Cardiovasculaires & CIC de Lyon Hôpital Louis Pradel, Hospices Civils de Lyon Lyon France; ^5^ Laboratoire d'Immunologie et Allergologie CHU d'Angers Angers France

**Keywords:** cardioprotection, clusterin, extracellular histones, inflammation, myocardial infarction

## Abstract

**Background:**

Acute myocardial infarction (AMI) remains one of the leading causes of mortality worldwide. Recently, a cardioprotective effect of clusterin (CLU), a ubiquitous extracellular chaperone, has been reported. However, the underlying mechanisms remain unresolved. We hypothesized that CLU exerts its protective effect on AMI by neutralizing cytotoxic and proinflammatory properties of extracellular histones, a new class of damage‐associated molecular patterns (DAMPs), that are released after massive cell injury.

**Methods and Results:**

In vitro, we showed that exogenous CLU reduces histone‐induced cell death in H9C2 cells after hypoxia−reoxygenation (.78 ± .15 vs. 1.39 ± .20; *p* = .0059). Moreover, we found increased CLU protein levels in the ischemic zone vs. non‐ischemic zone after AMI in mice (*p* < .05). Correspondingly, CLU‐deficient (CLU^−/−^) mice presented significantly increased infarct size vs. wild‐type (CLU^+/+^) mice (46.29 ± 5.13% vs. 27.47 ± 1.92%; *p* = .0176). This cardioprotective effect of CLU is accompanied by an attenuation of the post‐AMI proinflammatory response through a decrease in the expression of proinflammatory cytokines interleukin (IL)‐6 and IL‐1β, a decrease in phosphorylated nuclear factor kappa B (NF‐kB) p65, as well as a decrease in the activation of the nucleotide‐binding oligomerization domain (NOD)‐like receptor pyrin domain containing 3 (NLRP3) inflammasome. Also, we found that in patients with acute ST‐segment elevation myocardial infarction (STEMI), circulating CLU‐histone complexes were significantly increased compared to healthy controls (*p* < .001).

**Conclusions:**

From these results, CLU protects the heart from inflammatory injury in AMI and this cardioprotection is due at least in part to its ability to neutralise extracellular histones released from the damaged tissue.

## Graphical abstract

In vivo, CLU^−/−^ mice were more susceptible to acute myocardial infarction than CLU^+/+^ mice. Moreover, inflammation and histone levels were significantly increased in CLU^−/−^ mice compared to CLU^+/+^ mice following ischemia/reperfusion. In vitro, CLU protects cardiomyocytes from histone‐ and/or hypoxia‐induced cell death. CLU also reduces hypoxia‐induced pro‐inflammatory cytokine expression. In STEMI patients, the levels of CLU‐histone complexes were significantly increased.

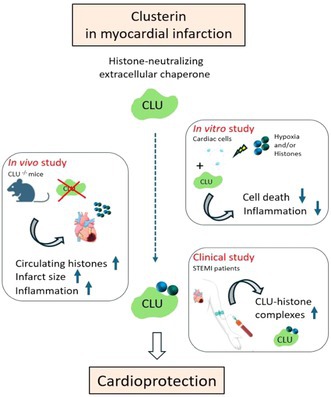

## 1 INTRODUCTION

Acute myocardial infarction (AMI) remains a leading cause of morbidity and mortality worldwide.^1^, ^2^ While prompt reperfusion of the myocardium is essential to limit excessive cardiomyocyte death, it can itself induce myocardial damage, a process called reperfusion injury.^3^

Inflammation is a hallmark of AMI and reperfusion injury.^4^ AMI triggers an intense local and systemic inflammatory response, which leads to the deterioration of cardiac function. There is increasing evidence that inflammation induced by myocardial ischemia‐reperfusion (IR) contributes to cardiomyocyte death.^5^ Thus, it is important to find new cardioprotective strategies to limit inflammation and ameliorate the prognosis of AMI patients. During AMI, dying cells release endogenous danger molecules called damage‐associated molecular patterns (DAMPs).^6^ Endogenous DAMPs are major drivers of sterile inflammation which, in turn, exacerbates myocardial tissue damage leading to the deterioration of cardiac function.^7^

Extracellular histones have emerged as pivotal mediators of tissue injury in a variety of diseases associated with severe tissue damage, including AMI.^8^ Released by dying cells, histones exhibit potent inflammatory and cytotoxic properties.^9^, ^10^ Based on these observations, extracellular histones are recognized as therapeutic targets of specific interest for treating AMI patients.

Clusterin (CLU), also known as apolipoprotein J, is a highly conserved extracellular chaperone constitutively expressed in almost all mammalian tissues and secreted in a variety of biological fluids.^11^ CLU is involved in multiple physiological processes including cell differentiation, cell‐cycle regulation, deoxyribonucleic acid (DNA) repair, transcription, apoptosis, lipid transport, tissue remodelling, and proteostasis.^12^, ^13^, ^14^ Under cell stress and tissue injury, CLU is up‐regulated^15^ and exerts anti‐inflammatory and cytoprotective effects.^16^ Studies have reported increased CLU levels in the heart and plasma early after myocardial infarction (MI),^13^ and in the left ventricle (LV) of heart failure patients, associated with left ventricular remodelling.^17^ CLU has been shown to protect cardiac cells from IR after heart transplantation.^18^ In animal studies, CLU is protective against myocardial IR.^19^ Interestingly, CLU has also been associated with a reduction in pro‐inflammatory signals.^18^ Nevertheless, the molecular mechanism of CLU‐mediated cardioprotection has not been fully elucidated. As a chaperone, CLU has been shown to interact with a broad range of proteins, including histones accumulated at the surface of dying cells.^20^

The aim of this study was to investigate the role of CLU in myocardial IR injury by different approaches, including in vivo AMI in mice, in vitro hypoxia‐reoxygenation (HR) in the H9C2 cell line and evaluation of the status of CLU in a cohort of STEMI patients. We sought to investigate the molecular mechanisms of CLU‐induced cardioprotection by evaluating the hypothesis that CLU, as a protein chaperone, is capable of attenuating histone‐induced myocardial damage.

## 2 MATERIALS AND METHODS

### 2.1 In vitro H9C2 HR

**FIGURE 1:**
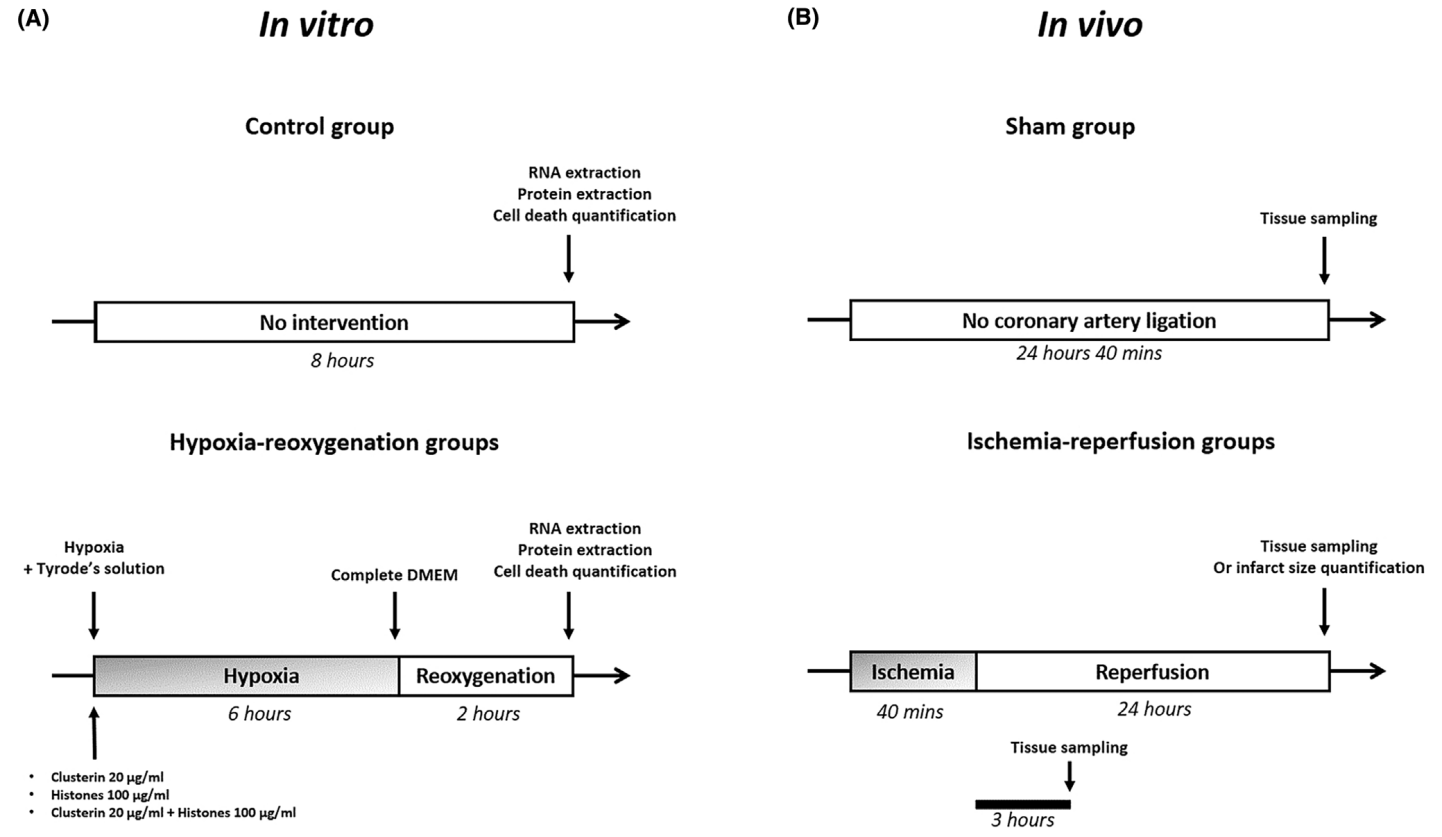
Schematic experimental procedures. In vitro and in vivo experimental design. (A) H9C2 cells, not treated or subjected to 6 h hypoxia followed by 2 h reoxygenation, were either treated with vehicle (PBS), 20 μg/mL CLU, 100 μg/mL histones, or with both during hypoxia. Cell death quantification, as well as RNA and protein expression, were analysed 2 h after reoxygenation. (B) Mice from the IR groups were subjected to 40 min coronary occlusion followed by 24 h reperfusion for infarct size assessment, and tissue sampling was performed after 3 and 24 h. Sham mice underwent no coronary artery ligation.

We used the rat cardiomyoblast cell line H9C2 in an in vitro HR model. Cells were cultured in Dulbecco's Modified Eagle Medium (DMEM) containing 4.5 g/L glucose and supplemented with antibiotics and 10% fetal calf serum (FCS). Cells were seeded in 12‐well plates. Hypoxia was induced by replacing culture medium by a glucose‐ and serum‐free isotonic solution (Tyrode's solution [mM]; sodium chloride (NaCl) 130, potassium chloride (KCl) 5, Hepes 10, magnesium chloride (MgCl_2_) 1, calcium chloride (CaCl_2_) 1.8; pH 7.4). Cells were placed into a hypoxia chamber (Adelbio, Clermont‐Ferrand, France) for 6 h at 37°C (Figure 1A). Hypoxia was obtained by flushing a stream of gas composed of 95% dinitrogen or nitrogen (N_2_) and 5% carbon dioxide (CO_2_) until the oxygen rate reached a value between .5% and 1%. Reoxygenation was achieved by replacing Tyrode's solution with culture medium in a standard incubator under normoxic conditions for 2 h. Cells were subjected to HR in the absence or presence of 100 μg/mL calf thymus histones (Sigma‐Aldrich, St Louis, MO) and without or with 20 μg/mL recombinant human CLU (R&D Systems, Minneapolis, MN); histone concentration was determined based on its toxicity on H9C2 cells subjected to HR (data not shown). Histones and CLU were added at the time of hypoxia in Tyrode's solution and present throughout the HR sequence. Cells were treated with histones alone for 8 h.

### 2.2 Cell death assessment

At the end of reoxygenation (i.e. 2 h), cells were collected and cell death was quantified by flow cytometry using 5 μg/mL propidium iodide (PI) (Sigma Aldrich); fluorescence was analysed using the MACSQuant flow cytometer (Miltenyi Biotec, San Diego, CA). A total of 10,000 events were acquired for each sample. Results were analysed using the FlowJo software and expressed as relative cell death relative to that in the non‐treated HR group.

### 2.3 Myocardial infarction model

All experimental procedures conform to the European Parliament Directive 2010/63/European Union (EU) and were approved by the regional ethics committee (Comité d'Ethique en Experimentation Animale des Pays de la Loire) and by the French Ministry of Higher Education and Research (agreement APAFIS 14109–2018031514075152 v4). The study was conducted in accordance with the ARRIVE 2.0 (Animal Research: Reporting of In Vivo Experiments) guidelines for animal research. 4–6‐week‐old C57BL6/J, CLU‐deficient (CLU^−/−^, global knockout mice, *n* = 14) and wild‐type (CLU^+/+^, *n* = 11) mice were anaesthetised by intraperitoneal (i.p.) injection of sodium pentobarbital (80 mg/kg; Ceva Santé Animale, Libourne, France) and ventilated via endotracheal intubation on a rodent ventilator (Minivent type 845; Harvard Apparatus, Holliston, MA). Body temperature was maintained at a constant 37°C using a homeothermic warming blanket (Harvard Apparatus). Mice received an injection of 1000 IU/kg heparin (heparin Choay1; Sanofi, Gentilly, France) prior to thoracotomy. Following skin incision, the chest was opened using a left lateral thoracotomy and the pericardium was removed. Coronary ligation was performed using a 7–0 prolene monofilament suture (Ethicon, Rarican, NJ) placed around the left coronary artery 1.5 mm under the tip of the left auricle and passed through a short piece of polyethylene tubing in order to create a reversible snare, as previously described.^21^, ^22^ Coronary occlusion was initiated by clamping the snare onto the epicardial surface directly above the coronary artery. Ischemia was confirmed through observations of dyskinesia and epicardial cyanosis of the myocardial region below the suture. For myocardial infarct size, mice underwent 40 min coronary artery occlusion followed by 24 h reperfusion (Figure 1B). For inflammatory markers monitoring, mice underwent 3 h reperfusion. After 40 min coronary occlusion, reperfusion was achieved by loosening the snare and confirmed by a marked hyperemic response at the time of reperfusion. For the sham group, animals underwent the same surgical procedure without coronary artery ligation. Animals were euthanised by means of exsanguination by excision of the heart under deep anaesthesia (sodium pentobarbital, 100 mg/kg i.p.). Immediately after excision, LV tissue samples from ischemic zone (IZ) or non‐ischemic zone (NIZ) were freeze‐clamped, or the heart was cut into slices for infarct size assessment.

### 2.4 Infarct size assessment

At the end of the 24 h reperfusion period, coronary ligation was repeated using the monofilament suture kept in place, and 400 μL Evans Blue 4% (Sigma‐Aldrich) was injected through the left‐ventricular apex, colouring the perfused (non‐ischemic) myocardium blue while the area at risk (AAR) remained unstained. The area‐at‐risk (AAR) was then identified as the non‐blue myocardium area and expressed as a percentage of LV area. The LV was cut into six to seven equal slices and incubated with 1% 2,3,5‐triphenyltetrazolium chloride pH 7.4 (TTC; Sigma‐Aldrich) at 37°C for 4 min to distinguish non‐viable (pale) from viable (red) myocardium. Slices were subsequently fixed in 10% formalin, thereby delimiting the viable myocardium in red and area of necrosis (AN) in pale white. Quantification of infarct size (planimetry) was performed blindly using ImageJ 1.47 software and expressed as a percentage of the area at risk (AN/AAR) and AAR as a percentage of total ventricular area (AAR%LV). Operators were blinded during both myocardial infarction surgeries and data analysis.

### 2.5 Analysis of CLU, nucleosomes and CLU‐histone complexes

Human and mouse CLU were quantified in serum using a commercial enzyme‐linked immunosorbent assay (ELISA) (R&D Systems, Abingdon, UK). Levels of nucleosomes in the serum and cell culture medium were determined using the Cell Death Detection ELISA^PLUS^ assay (Roche Diagnostics, Mannheim, Germany). Complexes formed by the interaction of CLU with histones H3 and H4 were evaluated in plasma using a homemade immunoassay with an anti‐human CLU monoclonal antibody (mAb) as capture Ab (R&D Systems) and horseradish peroxidase (HRP)‐conjugated anti‐H3 and anti‐H4 pAbs as detection Abs (Abcam, Cambridge, UK). Briefly, 96‐well plates (MaxiSorp®; Nunc, Roskilde, Denmark) were either uncoated or coated (overnight at 4°C) with 5 μg/mL anti‐CLU mAb (100 μL/well). After saturation with 5% bovine serum albumin (BSA) for 2 h, plates were incubated for 24 h at room temperature with plasma (150 μL/well) diluted 1:2 in phosphate buffered saline (PBS) containing 1% (w/v) BSA and .05% (v/v) Tween 20 and containing HRP‐labelled anti‐H3 and anti‐H4 Abs (1/1000 final dilution). After washing, bound Abs were detected using the chromogenic substrate 3,3,5,5′‐tetramethyl‐benzidine (TMB). Results are expressed as optical density (OD) values after subtraction of the OD value obtained with uncoated wells.

### 2.6 Western blot analysis

Western blots were performed on total protein extracts from either freeze‐clamped LV myocardium or H9C2 cell lysates, as described.^23^ Briefly, 15–75 μg total proteins were separated on a 10%–15% sodium dodecyl sulfate‐polyacrylamide gel electrophoresis (SDS‐PAGE) gel and transferred to a nitrocellulose membrane (Amersham Biosciences, Amersham, UK). After the nonspecific binding sites were blocked with 5% non‐fat milk for 1 h in Tris‐buffered saline Tween 20 (TBST), the membranes were incubated overnight at 4°C with rabbit anti‐CLU (1/1000; R&D Systems), anti‐phosphorylated (p‐)p65 (1/1000; Cell Signalling Technology, Danvers, MA), anti‐p65 (1/1000; Cell Signalling Technology) or anti‐GAPDH (1/10000; Sigma Aldrich) antibodies. After washing in TBST, the membranes were incubated for 1 h at room temperature with HRP‐labelled anti‐rabbit or anti‐mouse IgG antibodies (1/5000; Thermo Fisher Scientific, Waltham, MA). Bound antibodies were detected by chemiluminescence (Santa Cruz Biotechnologies, Santa Cruz, CA). The band densities with appropriate molecular mass (80 kDa for CLU, 65 kDa for p65) were determined semi‐quantitatively using Chemidoc imaging system (Biorad, Hercules, CA).

### 2.7 Reverse transcription quantitative polymerase chain reaction (RT‐qPCR)

The expression of interleukin (IL)‐6, nucleotide‐binding oligomerization domain (NOD)‐like receptor pyrin domain containing 3 (NLRP3), IL‐1β, and IL‐18 mRNA in H9C2 cells and LV tissues was determined by RT‐qPCR with hypoxanthine phosphoribosyltransferase (hprt), glyceraldehyde‐3‐phosphate dehydrogenase (gapdh) and glucuronidase beta (gusb) as references. Total RNA was extracted using the RNeasy Mini Kit, and the cDNA was synthesized using the Quantitect Reverse Transcription Kit following the manufacturer instructions (Qiagen, Hilden, Germany). RT‐qPCR was performed in a total volume of 20 μL reaction containing 10 ng cDNA and the SYBR™ Select Master Mix (Thermo Fisher scientific); PCR was performed using the Lightcycler®480 II Thermocycler (Roche Diagnostics). The thermal cycling conditions were as follows: 95°C for 3 min, followed by 40 cycles at 95°C for 15 s and 60°C for 1 min. The relative expression of target genes was calculated using the $2^{–\Delta \Delta C_{t}}$ method, normalized to the reference genes, and then compared with the control group in vitro or the NIZ in vivo. Primer sequences are listed in Table 1.

**TABLE 1:** Forward and reverse primer sequences for target and reference genes.

Genes	Species	Forward sequence	Reverse sequence
Gapdh	Mice	GACAATGAATACGGCTACAGCA	GGCCTCTCTTGCTCAGTGTC
Gusb	Mice	CTCTGGTGGCCTTACCTGAT	CAGTTGTTGTCACCTTCACCTC
Hprt	Mice	TGATAGATCCATTCCTATGACTGTAGA	AAGACATTCTTTCCAGTTAAAGTTGAG
Il‐1β	Mice	TGTAATGAAAGACGGCACACC	TCTTCTTTGGGTATTGCTTGG
Il‐6	Mice	CCAGGTAGCTATGGTACTCCAGAA	GATGGATGCTACCAAACTGGA
Nlrp3	Mice	TTTGTACCCAAGGCTGCTATCT	GTCTCGGGGCTTAGGTCCAC
Gusb	Rat	CTCTGGTGGCCTTACCTGAT	CAGACTCAGGTGTTGTCATCG
Il‐18	Rat	ACGGAGCATAAATGACCAAGTTC	TCTGGGATTCGTTGGCTGTT
Il‐6	Rat	GTATGTATGAACAGCGATGATG	CTCCAGGTAGAAACGGAACTC

### 2.8 Clinical study

The HIBISCUS‐STEMI (coHort of patients to Identify Biological and Imaging markerS of CardiovascUlar outcomes in ST elevation myocardial infarction) is a multicentric cohort composed of patients admitted for an acute STEMI. The trial design and protocol have been registered (NCT02823886; ClinicalTrials.gov). The cohort was approved by a local ethics committee (number: 2015‐067B). All patients gave written informed consent, and the study protocol conforms to the ethical guidelines of the 1975 Declaration of Helsinki. STEMI was defined according to the European Society of Cardiology guidelines by the presence of clinical symptoms (chest pain) associated with an ST‐segment elevation of more than 2 mm in two contiguous leads on a standard 12‐lead electrocardiogram, and significant troponin‐I elevation. Urgent reperfusion was achieved in all patients by primary percutaneous intervention (PCI) at admission. Main clinical and imaging characteristics of the study population among HIBISCUS‐STEMI cohort are presented in Table 2. Blood from HIBISCUS patients was collected at five time‐points: admission (H0), 4 h (H4), 24 h (H24), 48 h (H48), and 1 month (M1) after reperfusion. Plasma or serum was frozen at −80°C and stored at the Biological Resource Center of the Hospices Civils de Lyon (Lyon, France). Serum and plasma from healthy donors were from the blood collection center (EFS Pays de la Loire, Angers, France; agreement ANG‐2017‐01).

**TABLE 2:** Main clinical and imaging characteristics – HIBISCUS‐STEMI Cohort.

Male	33 (82.5)
Age (years)	56.3 [50.6; 61.4]
Body mass index (kg/m^2^)	25.99 [24.59; 29.16]
Hypertension arterial	8 (20)
Diabetes	2 (5)
Dyslipidemia	4 (10)
Current smoking	23 (57.5)
LAD occlusion	30 (75)
Onset to reperfusion (min)	120 [60; 191]
TIMI at admission = 0–1	21 (52.5)
Post‐PCI TIMI = 3	39 (97.5)
Infarct size (% of LV)	17.13 [7.95; 26.97]
Left ventricular ejection fraction (%)	53 [44; 58]
Left ventricular end‐diastolic volume (ml)	163.0 [146.5; 195.0]
Left ventricular end‐systolic volume (mL)	83.0 [62.7; 107.2]

*Note*: Values are expressed as number (percentage), or median IQR [25–75 percentiles].

Abbreviation: LAD, Left anterior descending artery.

### 2.9 Statistical analysis

Statistical analyses were performed using the GraphPad Prism 8.0.1. The sample size was calculated using G*Power software. After verifying data's distribution's normality using the Shapiro–Wilk test, the appropriate statistical test was performed. Levels of CLU, histones, and CLU‐histone complexes were identified as not normally distributed. Therefore, those variables were expressed as medians and interquartile range (IQR) and non‐parametric tests were used for comparison between groups. The Wilcoxon matched‐pairs signed rank test was used to compare STEMI patients at different points or mice, and the Kruskal–Wallis test was used to compare STEMI patients to healthy donors. Differences between two groups were evaluated using the Mann–Whitney *U* test. When the normal distribution is verified, one‐way analysis of variance (ANOVA) followed by post hoc Fisher's least significant difference (LSD) was performed for multiple group comparisons. Data are reported as mean ± standard error of the mean (SEM). All tests were two‐tailed, and a *p*‐value <.05 was considered significant.

## 3 RESULTS

### 3.1 Exogenous CLU reduces histone‐induced cell death during in vitro HR

**FIGURE 2:**
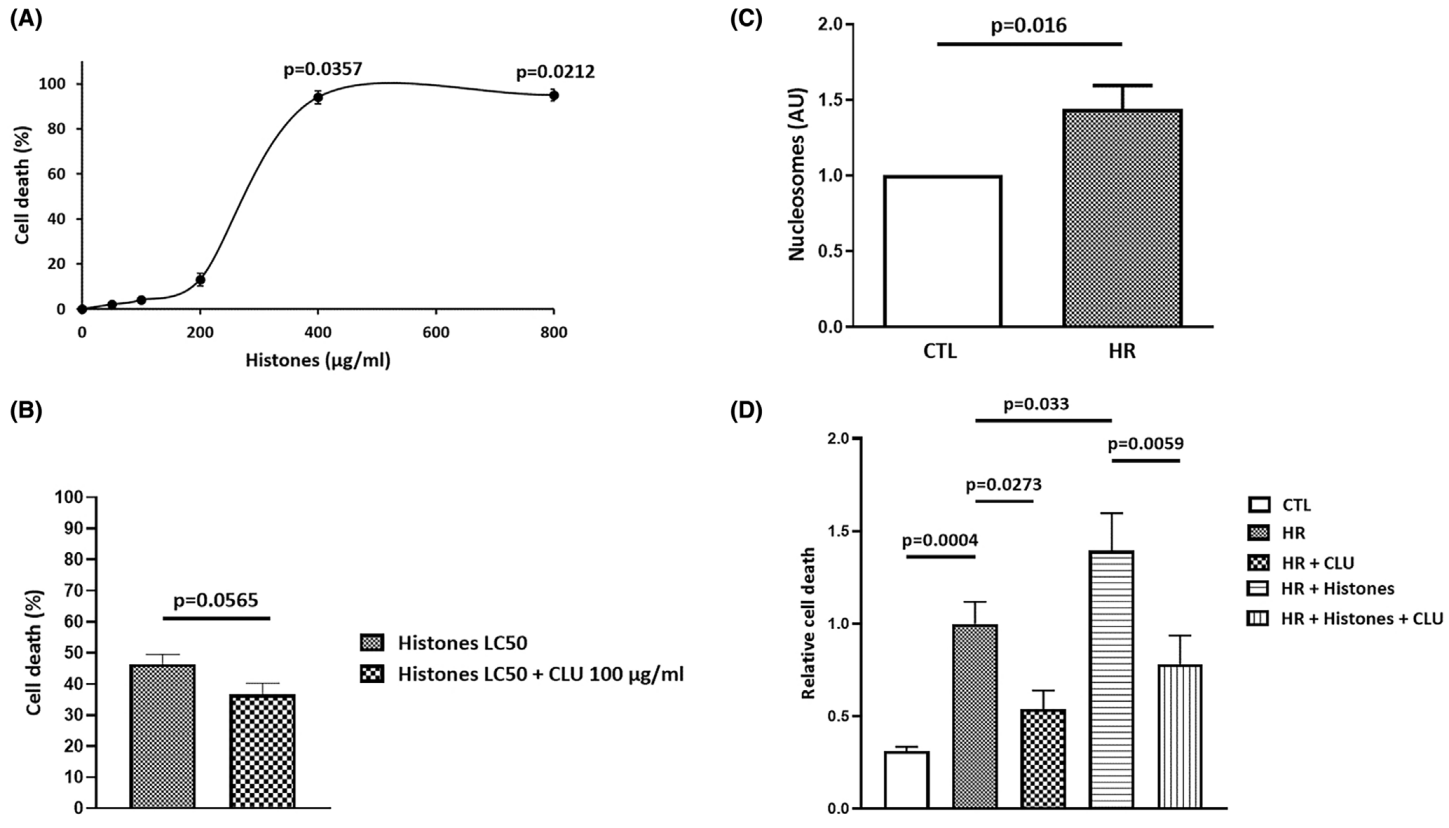
CLU protects cardiomyocytes from histone‐ or HR‐induced cell death. (A) The toxic effects of different histone concentrations on H9C2 cells (*n* = 3). Values are expressed as mean ± SEM. *p*‐values shown on the graph indicate statistical comparisons versus untreated cells. (B) CLU (100 μg/mL) in protecting H9C2 cells treated with 275 μg/mL histones (LC50) (*n* = 7). Values are expressed as mean ± SEM. (C) Measurement of nucleosome levels in the culture supernatants of H9C2 cells and HR cells (*n* = 8). Values are expressed as mean ± SEM. (D) Quantification of cell death in control (CTL), HR, HR + CLU, HR + histones, and HR + histones + CLU groups (*n* = 4–7). Values are expressed as mean ± SEM.

Extracellular histones are cytotoxic.^24^ We thus investigated whether CLU could protect cardiomyocytes from histone‐induced cell death. Figure 2A shows that calf thymus histones dose‐dependently induced H9C2 cell death; the concentration of histones inducing 50% cell death (lethal concentration 50 (LC50)) was LC50 = 275 μg/mL. The cytotoxic activity of 275 μg/mL histones was significantly reduced when 100 μg/mL CLU was added in the culture medium (46.43 ± 3.07% vs. 36.71 ± 3.53%, respectively; *p* = .0565) (Figure 2B). These data indicate that CLU can protect cardiomyocytes from extracellular histone‐induced cell death.

We then evaluated, in a model of HR, the release of histones by H9C2. After 6 h hypoxia followed by 2 h reoxygenation, we observed a significant release of nucleosomes in the culture medium (Figure 2C) that was associated with a significant increase in cell death in the HR group compared to controls (*p* = .0004; Figure 2D). We next investigated whether exogenous CLU may prevent HR‐induced cell death in H9C2 cells. Figure 2D shows that HR‐induced cell death was significantly inhibited by 20 μg/mL CLU added in the culture medium during hypoxia (relative cell death = .54 ± .10 vs. HR group; *p* = .0273), demonstrating that exogenous CLU protects H9C2 cells from HR.

Moreover, exogenous histones added to the culture medium during HR potentiated HR‐induced cell death (1.39 ± .20 vs. HR group; *p* = .0330) and this potentiating effect was also significantly reduced by the addition of CLU during HR (.78 ± .15 vs. 1.39 ± .20; *p* = .0059). These results confirmed that CLU is able to suppress the cytotoxic properties of extracellular histones in an in vitro cell‐based HR model.

### 3.2 CLU reduces HR‐induced pro‐inflammatory cytokine expression

**FIGURE 3:**
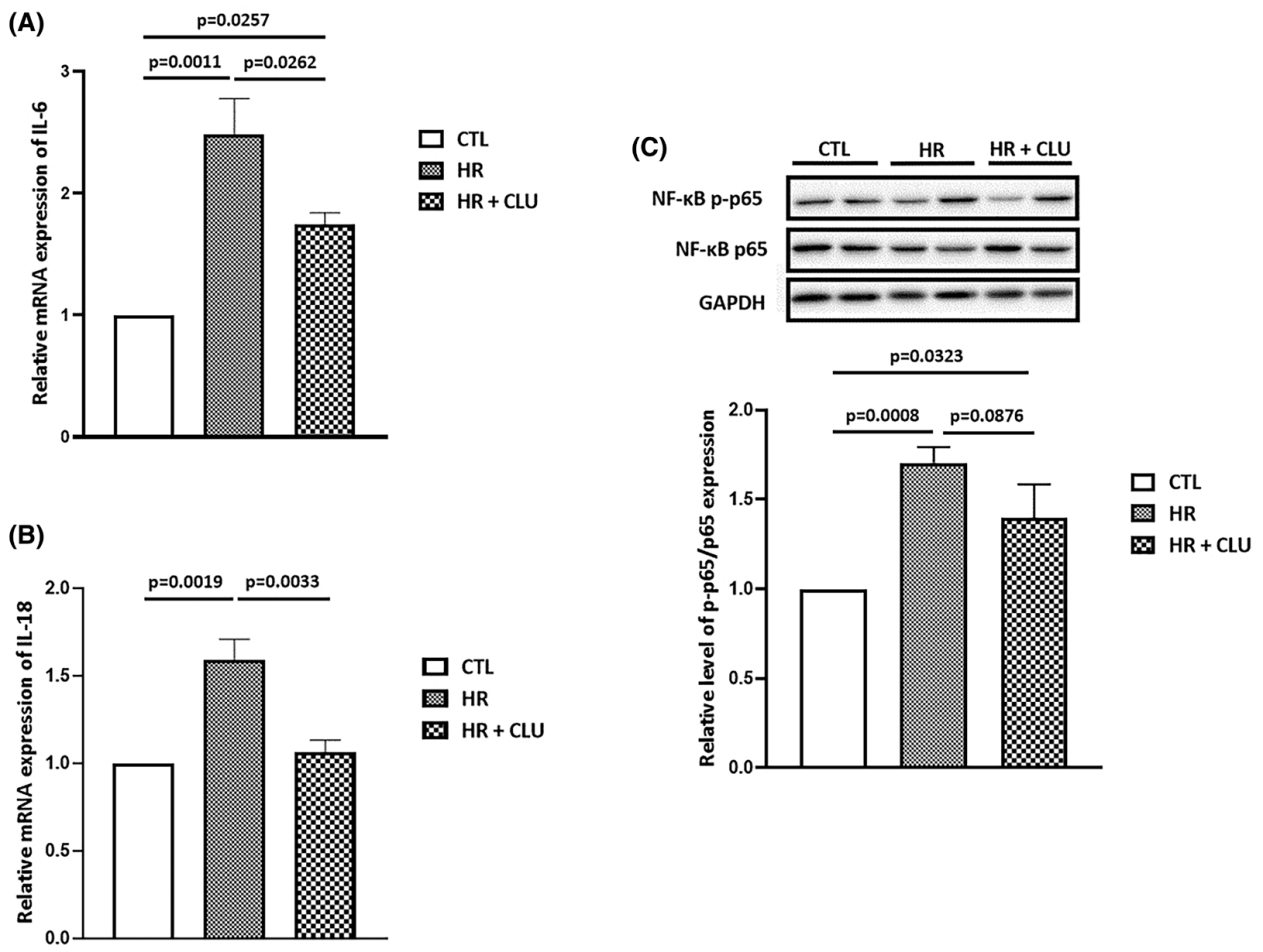
Inflammatory markers mRNA and protein expression, respectively by means of RT‐qPCR and western blot. IL‐6 (A), IL‐18 (B) mRNA expression in control (CTL), HR and HR + CLU samples (*n* = 3). (C) Representative immunoblots and histogram quantification of protein expression for NF‐κB p‐p65 and NF‐κB p65 in CTL, HR and HR + CLU groups (*n* = 6). Values are expressed as mean ± SEM.

HR induces the production of inflammatory cytokines.^25^ We then evaluated the capacity of CLU to modulate the expression of inflammatory mediators by H9C2 cells subjected to 6 h hypoxia followed by 2 h reoxygenation. IL‐6 and IL‐18 mRNA expression was significantly increased after HR, and this increase was significantly reduced in the presence of CLU (Figure 3A,B). The levels of phosphorylated nuclear factor kappa B (NF‐kB) p65, a transcription factor involved in inflammatory cytokine production, which expression was increased after HR, were reduced in the presence of CLU, although not reaching significance (Figure 3C). Collectively, these results indicate that CLU can reduce the inflammatory effects of HR in vitro.

### 3.3 CLU levels are increased following myocardial IR in mice

**FIGURE 4:**
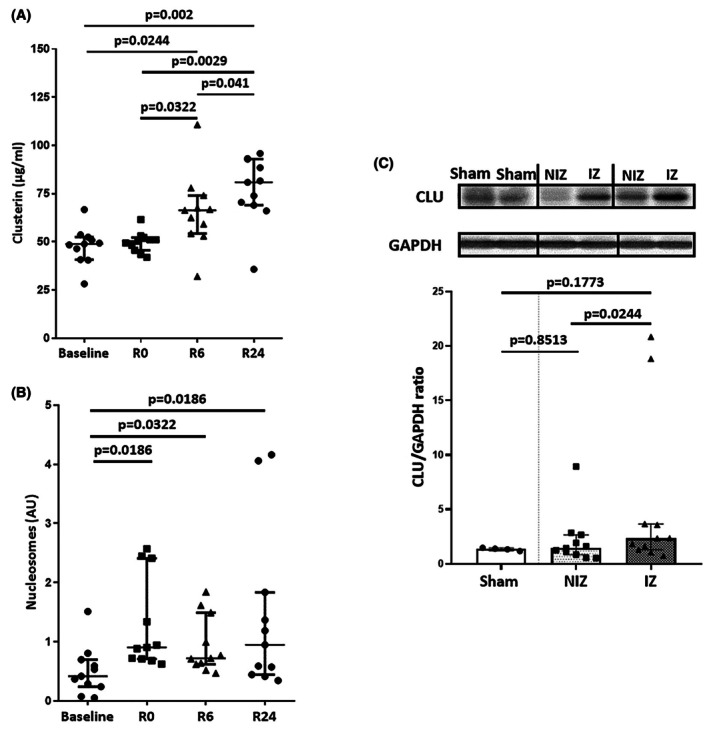
CLU levels are increased in C57BL6 mice. Both circulating levels of CLU (A) and nucleosomes (B) were quantified in mice at baseline (before ischemia), R0 (at the time of reperfusion), R6 and R24 (6 and 24 h, post reperfusion, respectively) (*n* = 11). (C) Representative immunoblots (upper panel) and histogram quantification (lower panel) of CLU in LV myocardial tissue from the sham group (*n* = 4) and in ischemic (IZ) and non‐ischemic zones (NIZ) after 24 h reperfusion (*n* = 11). GAPDH was used as a loading control. Values are expressed as median IQR (25–75 percentiles).

We next evaluated the levels of circulating CLU and nucleosomes in the serum of C57BL6/J mice subjected to 40‐min coronary artery ligation followed by 24‐h reperfusion. CLU levels were significantly increased at 6‐h and 24‐h post‐AMI compared to baseline (66.39 μg/mL IQR [54.29–74.00]; 80.80 μg/mL IQR [68.96–92.91]; vs. 48.83 μg/mL IQR [40.77–52.42]; *p* = .0244 and *p* = .002, respectively) (Figure 4A). Serum nucleosome levels were significantly increased at R0, R6, and R24 compared to baseline (arbitrary unit (AU) = .90 IQR [.71–2.41]; .72 IQR [.62–1.49]; .95 IQR [.44–1.83]; vs. .42 IQR [.24–.69]; *p* = .0186, *p* = .0322 and *p* = .0186, respectively) (Figure 4B).

We then measured CLU in LV myocardial tissues from sham mice and mice subjected to 40‐min myocardial ischemia followed by 24 h reperfusion. Results showed that the levels of CLU were significantly increased in IZ compared to NIZ (*p* < .05; Figure 4C).

### 3.4 CLU ^−/−^ mice are more susceptible than wildtype mice to myocardial IR

**FIGURE 5:**
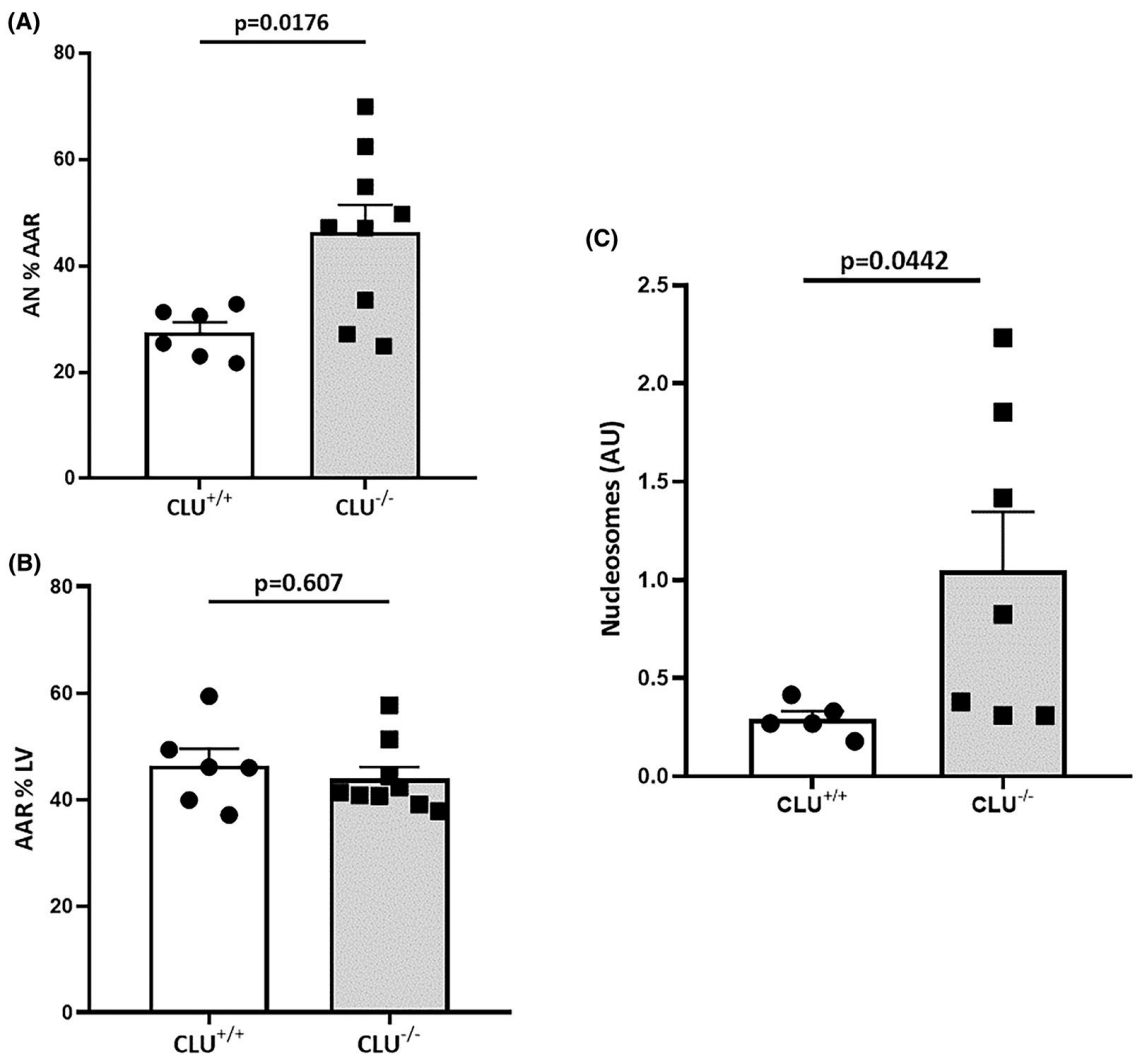
Susceptibility of CLU^−/−^ mice to myocardial IR. Scatter dot blot showing (A) area of necrosis (AN) as a percentage of area at risk (AAR), (B) AAR as a percentage of total LV area after 24 h reperfusion (*n* = 6–9) and (C) nucleosome levels after 24 h reperfusion. Values are expressed as mean ± SEM.

We evaluated the protective role of CLU in a mouse model of AMI. Results revealed that CLU^−/−^ mice were more susceptible to AMI than CLU^+/+^ mice (Figure 5A). Infarct size was significantly higher in CLU^−/−^ compared to CLU^+/+^ mice (46.29 ± 5.13% vs. 27.47 ± 1.92%; *p* = .0176), whereas AAR did not significantly differ between the two groups (43.94 ± 2.15% vs. 46.32 ± 3.2%; *p* = .670) (Figure 5B). Of note, the mice mortality rate induced by AMI was significantly higher in CLU^−/−^ compared to CLU^+/+^ mice (54% vs. 31%), further highlighting the protective role of CLU during AMI. We found increased nucleosome levels in CLU^−/−^ mice following IR (Figure 5C), supporting our hypothesis that CLU's cardioprotective effect involves its function as a chaperone for extracellular histones released post‐MI.

### 3.5 Pro‐inflammatory cytokines and NLRP3 inflammasome are upregulated in CLU ^−/−^ mice subjected to myocardial IR

**FIGURE 6:**
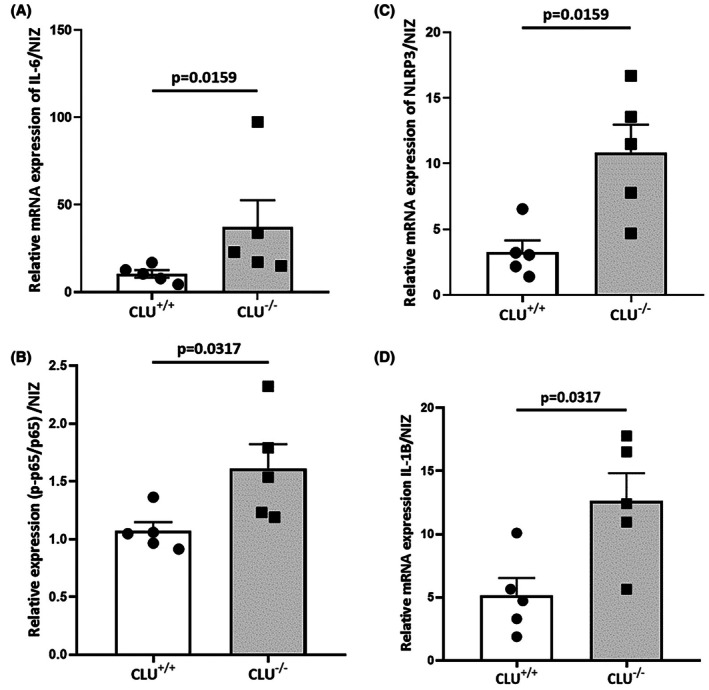
Inflammatory markers mRNA and protein expression, respectively, by means of RT‐qPCR and western blot. IL‐6 (A), NLRP3 (C) and IL‐1β (D) mRNA expression in LV samples collected from CLU^+/+^ and CLU^−/−^ mice 3 h after reperfusion (*n* = 5). (B) Histogram quantification of p‐p65 and p65 expression in LV samples from CLU^+/+^ and CLU^−/−^ mice collected 3 h after reperfusion (*n* = 5). Values are expressed as mean ± SEM.

To investigate the role of CLU during the inflammatory process associated with ischemia, the expression of NLRP3, IL‐1 and IL‐6 mRNA was evaluated in CLU^−/−^ and CLU^+/+^ mice 3 h after reperfusion. Results showed that the expression of the transcripts encoding IL‐6 (Figure 6A) and NF‐κB p65 (Figure 6B) was significantly increased in CLU^*−/−*^ mice compared to CLU^+/+^ mice. ^*−/−*^Moreover, the ischemic hearts of CLU^−/−^ mice exhibited enhanced inflammasome activation, as evidenced by an increase in NLRP3 and IL‐1β mRNA expression compared to CLU^+/+^ mice (Figure 6C,D).

### 3.6 Levels of CLU and CLU‐histone complexes in AMI patients

**FIGURE 7:**
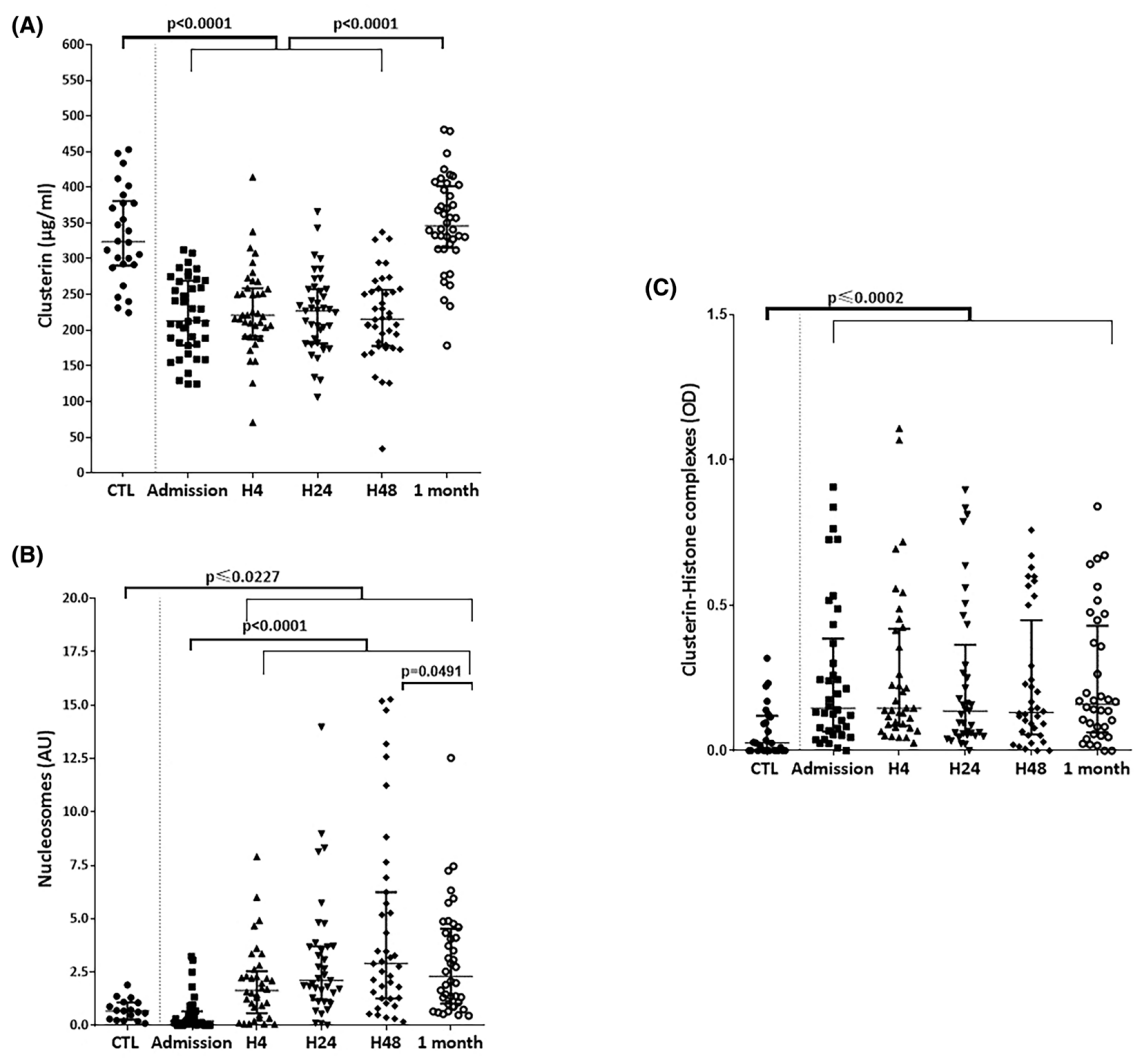
CLU levels are decreased in STEMI patients. Measurement of circulating levels of CLU (A), nucleosomes (B) and CLU‐histones complexes (C) in STEMI patients at different time points after coronary angioplasty (*n* = 36–40) and healthy subjects (CTL) (*n* = 17–29). Values are expressed as median IQR (25–75 percentiles). OD, Optical density.

In order to evaluate the role of CLU in the context of AMI in humans, we first quantified serum CLU levels in a cohort of 36–40 STEMI patients with acute coronary syndrome at five time‐points: admission (H0), 4 h (H4), 24 h (H24), 48 h (H48), and 1 month (M1) after revascularization (Figure 7A). After coronary angioplasty, serum CLU levels of STEMI patients (acute coronary syndrome with ST‐segment elevation) were significantly lower compared to healthy donors, at H0, H4, H24 and H48 after revascularization (323.9 μg/mL IQR [290.6–380.8]; 213.1 μg/mL IQR [178.7–269.3]; 220.8 μg/mL IQR [191.52–258.9]; 227.2 μg/mL IQR [181.3–257.4]; 215.6 μg/mL IQR [177.8–256.7] vs. 346.0 μg/mL IQR [315.4–401.6]: *p* < .0001) (Figure 7A). Interestingly, one month post‐AMI, circulating CLU levels were significantly increased compared to the early stage of AMI, reaching the levels found in healthy subjects (Figure 7A).

The levels of circulating nucleosomes reflect the severity of tissue damage,^26^ especially during AMI^8^ and reflect the levels of circulating histones. Serum nucleosome levels were significantly increased in STEMI patients at H4, H24 and H48 compared to healthy subjects (arbitrary unit (AU) = 1.63 IQR [.57–2.53]; 2.10 IQR [1.20–3.70]; 2.90 IQR [1.26–6.24]; vs. .68 IQR [.26–1.07]; *p* = .0227, *p* = .0004 and *p* < .0001, respectively) (Figure 7B). Nucleosome levels were significantly decreased 1 month after reperfusion compared to H48 (arbitrary unit (AU) = 2.30 IQR [1.02–4.53] vs. 2.90 IQR [1.26–6.24], *p* = .0491) (Figure 7B).

Finally, we evaluated the presence of CLU‐histone complexes in the plasma of STEMI patients and healthy subjects. The levels of CLU‐histone complexes were significantly increased in STEMI patients compared to healthy subjects (OD values = .15 IQR [.06–.38]; .15 IQR [.08–.42]; .14 IQR [.06–.36]; .13 IQR [.06–.45]; .16 IQR [.06–.43] vs. .03 IQR [.00–.12], respectively; *p* < .0001, *p* < .0001, *p* = .0002, *p* = .0002 and *p* < .0001, respectively) (Figure 7C). Interestingly, the levels of CLU‐histone complexes parallel circulating nucleosomes levels up to 48 h (Figure 7C).

## 4 DISCUSSION

In the present study, we sought to examine the cardioprotective role of the extracellular chaperone CLU in AMI and to explore the underlying cellular and molecular mechanisms. Using an in vitro model of HR, we showed that exogenous CLU is able to suppress the cytotoxic and pro‐inflammatory properties of extracellular histones on the cardiomyocyte cells H9C2. Moreover, we observed that CLU^−/−^ mice were more susceptible to myocardial IR injuries in vivo. In patients, we observed a decrease of CLU levels after AMI, which was associated with an increase in circulating histones and CLU‐histone complexes levels. These results suggest that CLU is likely to neutralize extracellular histones released by dying cells during AMI, therefore preventing histone‐induced myocardial damage.

The pathophysiological mechanisms underlying myocardial IR injury are multifactorial and may involve several factors, including inflammation. It is well known that an excessive inflammatory response during AMI can exacerbate injury, influencing infarct size and therefore increasing the risk of adverse remodelling and clinical outcome.^27^, ^28^ The inflammatory response during AMI involves two distinct phases: an initial acute pro‐inflammatory phase, which allows for the clearance of dead cells and debris from the damaged site, followed by an anti‐inflammatory phase which aims to promote tissue repair and restore cardiac function. Both phases are crucial for the healing process and have implications for cardioprotection. Therefore, effective cardioprotection requires a balance between adequate initial inflammation for debris clearance and robust anti‐inflammatory responses to promote healing. Understanding these phases is crucial for the development of targeted therapies that can modulate the inflammatory response to maximize cardioprotection and improve patient outcomes following AMI.

Extracellular histones released by dying cells have emerged as major DAMPs in several diseases, including sepsis,^29^ ischemic stroke^30^ and AMI^8^ and have been implicated in multiple organ injury pathogenesis.^31^ Myocardial IR induces an increase in circulating nucleosomes, which correlates with infarct size.^32^ Interestingly, released nucleosomes likely originate from neutrophil extracellular traps (NET)osis.^33^ Extracellular histones trigger sterile inflammation, probably via the toll‐like receptor (TLR)2/TLR4, TLR9 and NLRP3 pathways^34^, ^35^, ^36^, ^37^ and exert potent cytotoxic effects in cardiomyocytes in vitro.^8^, ^38^ Considering the critical role of inflammation during cardiac injury, their neutralization appears as an interesting therapeutic strategy in AMI. In agreement, different histone‐neutralizing molecules, such as heparin, albumin, pentraxin 3, and C‐reactive protein,^39^, ^40^, ^41^ attenuate histone cytotoxicity.^31^

CLU is an ubiquitous and multifunctional glycoprotein that is present in almost all tissues and biological fluids.^11^, ^15^ It plays numerous and sometimes opposite roles in a number of physiological and pathophysiological situations, which makes it an enigmatic protein. The secreted form of CLU is increased in response to a wide variety of stress signals, including IR injury. In brain IR injury, CLU has been identified as a marker of brain damage in patients undergoing carotid endarterectomy.^42^ In AMI, both increase or decrease of plasma CLU levels have been reported, depending on the stage of MI. Several studies have shown increased circulating CLU levels at the early stage of AMI.^14^, ^43^, ^44^ On the contrary, studies reported lower levels of CLU in AMI patients immediately after MI compared to healthy subjects.^45^ Elevated plasma CLU levels have also been reported in plasma of patients at late stage after MI that were associated with left ventricular remodelling.^17^ In a preclinical porcine model of reperfused AMI, plasma CLU levels have been shown to be increased 120 min after the onset of AMI before returning to normal levels after 3 days.^13^ Here, in STEMI patients, we found decreased levels of CLU that returned to normal levels at 1 month. Those reduced levels were associated with a parallel increase of circulating histone levels. The decrease of plasma CLU levels in STEMI patients may result from its binding to extracellular histones released by dying myocardial cells and their removal from circulation as CLU‐histones complexes. Cubedo et al reported that the proteomic profile of CLU was altered in AMI patients, with a differential glycosylation pattern.^45^

The in vivo protective role of CLU was confirmed in a murine model of AMI. Indeed, we noticed a higher mortality of CLU^−/−^ mice compared to CLU mice^+/+^ following AMI, suggesting that the capacity of CLU to neutralise histones was overwhelmed, leaving histones available to exert their cytotoxicity.

We showed that the levels of CLU were upregulated in the ischemic area of infarcted mice. Interestingly, CLU serum levels were also found elevated in mice 24 h after reperfusion. Our results are in accordance with earlier studies reporting increased expression of CLU in the infarct and border tissue zones after AMI.^43^, ^44^, ^46^ Interestingly, the recent study by Li et al. showed that the expression of clusterin decreased in myocardium in an infarct time‐dependent manner in mice subjected to AMI.^47^ Even though the nature of CLU‐expressing cells remains to be elucidated, one can argue that this increase may result from a local synthesis of CLU by cardiomyocytes in response to ischemia (tissue injury). Indeed, Krijnen et al showed that CLU was rapidly expressed in response to ischemia in H9C2 cells.^48^ Alternatively, Väkevä et al, reporting CLU deposits in infarcted regions of human myocardium, suggested that CLU could originate from circulation following reperfusion as a result of diffusion from plasma in infarcted lesions.^44^

Several lines of evidence suggest that CLU is cytoprotective^15^ and anti‐inflammatory.^16^ In H9C2 cells under hypoxic conditions, Li et al showed the treatment with clusterin significantly increased cell viability.^47^ These findings are in accordance with our results showing that clusterin protects H9C2 cells from HR‐induced cell death. However, the exact mechanism whereby CLU protects injured cardiomyocytes is not yet clear. Following cardiac transplantation, CLU reduces the expression of the inflammatory cytokines IL‐1β and TNFα induced by a stress signal through inhibition of the NF‐κB signalling pathway, thereby reducing apoptosis and IR injury.^18^, ^49^ CLU has also been shown to protect cardiomyocytes against hydrogen peroxide (H_2_O_2_)‐induced oxidative stress through activation of the protein kinase B (Akt)/glycogen synthase kinase‐3 beta (GSK‐3β) signalling pathway.^50^ In infarcted human heart, CLU was found to colocalize with complement on jeopardized human cardiomyocytes after AMI.^44^ CLU was also found to protect cardiomyocytes against ischemic cell death in a complement‐independent pathway.^48^

CLU is thought to function as an extracellular chaperone which binds to a wide variety of ligands, including misfolded or damaged proteins, toxic molecules, components of the complement membrane attack complex, or lipids.^11^ CLU also binds to cellular debris, thereby protecting cells from debris‐inflicted damage.^51^ However, little is known on the mechanism allowing CLU to exert its chaperone activity, such as stabilization of damaged proteins. CLU forms stable, soluble high‐molecular‐weight complexes with misfolded client proteins.^52^ One can hypothesize that CLU may act as a scavenger of extracellular histones. This hypothesis is supported by a previous study reporting that CLU binds to histones accumulated at the surface of dying cells. CLU would interact with hydrophobic domains of histones^20^ through its amphipathic a‐helices with higher hydrophobic moments.^53^ In our recently published paper,^54^ we showed that clusterin binds to circulating histones and neutralises their inflammatory, thrombotic and cytotoxic properties both in vitro and in vivo (experimental sepsis). Specifically, clusterin binds to the histones H2A, H2B, H3 and H4 as well as to total histones, whereas there was no interaction with other danger molecules released by dying cells, such as calreticulin, heat shock protein 70 (HSP70), genomic DNA or high mobility group box‐1 (HMGB1). Our present study supports the potential of CLU as a novel and promising cardioprotective agent against ischemia/reperfusion injury. Given the persistent lack of effective therapies for acute myocardial infarction, we believe that targeting inflammation through CLU‐based approaches may offer significant clinical benefits. Our findings suggest that restoring CLU levels, potentially via exogenous administration, may represent an effective therapeutic strategy for AMI patients, in which extracellular histone release from injured myocardial tissue exacerbates inflammation and tissue damage. By mitigating histone‐driven inflammation, CLU supplementation may help improve AMI management. Moreover, CLU has been suggested as a potential biomarker for various conditions, including cardiovascular diseases.^17^, ^45^ In the context of AMI, the clinical translation of CLU as a biomarker could be significant. Clusterin could potentially serve as a biomarker for early diagnosis or prognosis, helping clinicians in identifying patients at risk of adverse outcomes post‐AMI. Further studies are needed to fully elucidate the role of CLU in this context and to support its potential clinical translation.

Here we show, for the first time, that CLU^−/−^ mice are more susceptible to myocardial IR than CLU^+/+^ mice. This cardioprotective effect is accompanied by a decrease in the local pro‐inflammatory response at the early phase of reperfusion. Our data indicate that a loss of CLU expression results in an increase in inflammation and exacerbates mice susceptibility to IR injury. We also showed an increase in the inflammatory cytokine IL‐1β in the ischemic area of CLU^−/−^ mice at the early phase of reperfusion, reflecting the activation of the NLRP3 inflammasome and its involvement in the setting of IR injury in CLU^−/−^ mice. Similarly, a recent study showed that CLU overexpression attenuates cholesterol crystal‐induced inflammation through inhibition of NLRP3 while preventing the production of TNFα, IL‐1β, and IL‐18.^55^ Accordingly, the administration of CLU in rats subjected to AMI was protective.^19^ Inflammation could represent a key mechanistic link between clusterin and myocardial infarction. Here we found that CLU^−/−^ mice are more susceptible to myocardial I/R injury. Thus, the enhanced infarct size we found in CLU‐deficient mice is likely to result from enhanced inflammation due to the absence of CLU to neutralize histones. Clusterin has been increasingly recognised for its role in various inflammatory conditions,^56^ and it possesses well‐described immunomodulatory and cytoprotective properties. On the other hand, acute myocardial infarction is known to trigger a strong sterile inflammatory response that contributes to further myocardial damage,^57^, ^58^ largely mediated by the release of damage‐associated molecular patterns (DAMPs) from necrotic cardiomyocytes, including extracellular histones. Given its chaperone‐like functions, CLU may play a protective role by binding to and neutralising extracellular histones, thereby limiting their cytotoxic and pro‐inflammatory effects. Reduced levels of CLU during the acute phase may reflect both its consumption in response to inflammation and a potential limited capacity to counteract ongoing damage. Therefore, a relative deficiency or rapid consumption of CLU in the acute phase of myocardial infarction could contribute to insufficient control of inflammation, potentially exacerbating myocardial damage. This hypothesis aligns with our findings and supports the notion that inflammation is a central mediator connecting CLU levels and myocardial injury. Taken together, our data indicate a cardioprotective role of CLU in AMI, probably through its unique capacity to neutralise extracellular histones released by injured tissues. As a proof of concept that the increased susceptibility to myocardial ischemia/reperfusion observed in global CLU knockout mice is due to lack of histone neutralisation, future studies should evaluate whether neutralising histone (using agents such as heparin or polysialic acid) could reverse the phenotype in mice lacking CLU.

## 5 LIMITATIONS

We used an in vitro HR model to mimic myocardial IR injury. Certainly, the reported findings in vitro cannot be directly translated to a clinical setting, though it represents a substantial limitation of this study. One of the main limitations of this study is the use of undifferentiated cardiomyoblasts rather than primary cardiomyocytes to explore in vitro the mechanisms underlying the protective effect of CLU.^59^ Thus, additional efforts are needed to explore whether the protective functions of CLU under hypoxic conditions can be reproduced in adult primary cardiomyocytes to ascertain the cytoprotective effects of clusterin. Potential confounding factors such as pharmacological treatments and clinical heterogeneity among STEMI patients, including age, sex, comorbidities, may have influenced the observed outcomes. Future studies with larger cohorts will be important to validate these findings and assess their broader applicability.

## AUTHOR CONTRIBUTIONS

All authors contributed to the study conception and design. ST, FP, and LA designed the study. LA, AP, ST, PP, JB, and LL conducted experiments. LA, ST, JB, PP, and AP analysed the data. ST and LA drafted the manuscript. YD, PJ, FP, CB, and DH revised the manuscript. All authors read and approved the final version of the manuscript. ST and FP are co‐last authors based on their equal contribution.

## FUNDING INFORMATION

This work was supported by Angers University Hospital.

## CONFLICT OF INTEREST STATEMENT

The authors declare that they have no conflict of interest. This is an original manuscript and has not been previously published or submitted to any other journal.

## Data Availability

The data that support the findings of this study are available from the corresponding author upon reasonable request.
